# Supplementary material for: The Small RNA Universe of Capitella teleta
Source: Front Mol Biosci. 2022 Feb 25;9:802814. doi: 10.3389/fmolb.2022.802814 (PMC8915122; doi:10.3389/fmolb.2022.802814)
Supplement: Supplementary file 1 [file DataSheet1.ZIP › Supplement/known/CAPTEscaffold_910_30369.pdf]

Provisional ID	:	CAPTEscaffold_910_30369
Score total	:	188635.4
Score for star read(s)	:	3.9
Score for read counts	:	188625.2
Score for mfe	:	1.7
Score for randfold	:	1.6
Score for cons. seed	:	3
Total read count	:	369992
Mature read count	:	367921
Loop read count	:	0
Star read count	:	2071

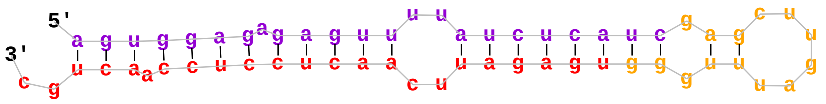

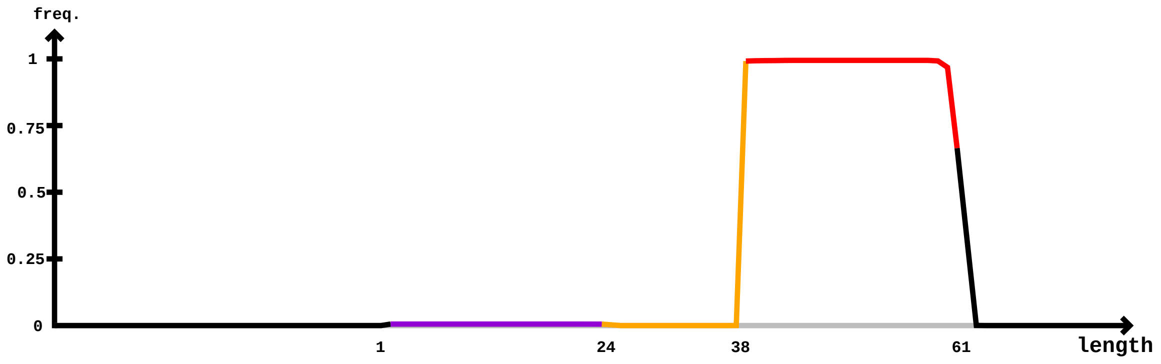

Star Mature

```
5'- uuucguucuuuuccguuugcggugcuugagauuaguggagagaguuuuaucucaucgagcuugauuuggg ugagauucaacuccuccaacugcucucacacugaaauuugc -3'   obs
    uuucguucuuuuccguuugcggugcuugagauuaguggagagaguuuuaucucaucgagcuugauuuggg ugagauucaacuccuccaacugcucucacacugaaauuugc        exp
    uuucguucuuuuccguuugcggugcuugagauuaguggagagaguuuuaucucaucgagcuugauuuggg ugagauucaacuccuccaacugcucucacacugaaauuugc        known
    ....................(((((..(((((.((((((((.(((((..((((((((.((......)).))))))))..))))))))).)))).)))))))))).......   reads   mm   sample
    ...........uccguuugcggugcuugagauuaguggaga......................................................................   1   0   seq
    .................ugcggugcuugagauuaguggagagaguuu................................................................   1   0   seq
    ......................ugcuugagauuaguggagagaguuuuaucu...........................................................   2   0   seq
    ..........................ugagauuaguggagagaguuuuaucucaucg......................................................   1   0   seq
    ................................Caguggagagaguuuuaucucauc.......................................................   1   1   seq
    .................................aguggagagaguuuuauc............................................................   1   0   seq
    .................................aguAgagagaguuuuaucu...........................................................   1   1   seq
    .................................aguggagagaguuuuaucu...........................................................   1   0   seq
    .................................aguggagagaguuuuaucuc..........................................................   32   0   seq
    .................................aguggagagaguuuuaucuca.........................................................   12   0   seq
    .................................aguggagagaguuuuaucucau........................................................   24   0   seq
    .................................aguggagagaguuuuaucuAau........................................................   1   1   seq
    .................................aguggagagaguuuuaGcucauc.......................................................   1   1   seq
    .................................aguggagagaguuuuaucucauA.......................................................   3   1   seq
    .................................aguggagagaguuuuaucuAauc.......................................................   2   1   seq
    .................................aguggGgagaguuuuaucucauc.......................................................   1   1   seq
    .................................Nguggagagaguuuuaucucauc.......................................................   1   1   seq
    .................................aguggagagaguuuuaucucauc.......................................................   1934   0   seq
    .................................aguggagaAaguuuuaucucauc.......................................................   1   1   seq
    .................................aguggagagaguuuuaucucauU.......................................................   4   1   seq
    .................................aguggagagaguuuuauAucauc.......................................................   1   1   seq
    .................................agugAagagaguuuuaucucauc.......................................................   2   1   seq
    .................................Gguggagagaguuuuaucucauc.......................................................   5   1   seq
    .................................agAggagagaguuuuaucucauc.......................................................   2   1   seq
    .................................aguggagagaguuuuaucucaCc.......................................................   1   1   seq
    .................................aguggUgagaguuuuaucucauc.......................................................   1   1   seq
    .................................aguAgagagaguuuuaucucauc.......................................................   10   1   seq
    .................................aguggagaCaguuuuaucucauc.......................................................   1   1   seq
    .................................aguggagagaguuuCaucucauc.......................................................   1   1   seq
    .................................aguggagagaguuuuaucuUauc.......................................................   3   1   seq
    .................................aguCgagagaguuuuaucucauc.......................................................   1   1   seq
    .................................aguUgagagaguuuuaucucauc.......................................................   1   1   seq
```

Star Mature

uuucguucuuuuccguuugcggugcuugagauuaguggagagaguuuuaucucaucgagcuugauuuggguga gauucaacuccuccaacugcucucacacugaauuugc			
..................................guggagagaguuuuaucucauc..	22	0	seq
....Cugagauucaacuccuccaacug..................	11	1	seq
....Cugagauucaacuccuccaacugc.................	44	1	seq
....Augagauucaacuccuccaacugc.................	3	1	seq
....ugagauucaacuccucca......................	2	0	seq
....ugagauucaacuccuccU......................	1	1	seq
....ugagauucaacuccuccaa.....................	22	0	seq
....uAagauucaacuccuccaac....................	2	1	seq
....ugagauucaacuccuccaaU....................	1	1	seq
....ugagauucaacucAuccaac....................	2	1	seq
....ugagauucaacuccuccaac....................	728	0	seq
....Agagauucaacuccuccaac....................	1	1	seq
....ugagauucaacuccucUaac....................	1	1	seq
....ugagauuUaacuccuccaac....................	1	1	seq
....ugagauucaacuAcuccaac....................	1	1	seq
....ugagauucGacuccuccaac....................	1	1	seq
....ugaCauucaacuccuccaac....................	1	1	seq
....ugagauuAaacuccuccaac....................	1	1	seq
....ugagauucaacuccucAaac....................	1	1	seq
....ugagauucGacuccuccaacu...................	4	1	seq
....ugagauucaacuccuccaCcu...................	1	1	seq
....ugagauucaacCccuccaacu...................	2	1	seq
....ugagauucaUcuccuccaacu...................	1	1	seq
....ugagauucaacuccuccaacu...................	8727	0	seq
....ugagauucaacAccuccaacu...................	2	1	seq
....ugagauucaacuccAccaacu...................	3	1	seq
....ugagauCcaacuccuccaacu...................	2	1	seq
....ugagauucaacuAcuccaacu...................	3	1	seq
....uAagauucaacuccuccaacu...................	9	1	seq
....ugagaCucaacuccuccaacu...................	16	1	seq
....Cgagauucaacuccuccaacu...................	4	1	seq
....ugagauucaacuccuccaaAu...................	2	1	seq
....ugagauucaacuccCccaacu...................	4	1	seq
....ugagauucaacucAuccaacu...................	8	1	seq
....ugagauucaacuccuccaacG...................	33	1	seq
....Agagauucaacuccuccaacu...................	20	1	seq
....ugagauucaGcuccuccaacu...................	5	1	seq
....ugagauucaacucUuccaacu...................	6	1	seq
....ugagauucaacuccuccaacA...................	14	1	seq
....ugagaAucaacuccuccaacu...................	1	1	seq
....ugaUauucaacuccuccaacu...................	1	1	seq
....ugagauucaacuccuccaUcu...................	1	1	seq
....uCagauucaacuccuccaacu...................	1	1	seq
....ugagauuAaacuccuccaacu...................	4	1	seq
....ugagauAcaacuccuccaacu...................	6	1	seq
....ugagauucaacuccuccaacC...................	3	1	seq
....ugagauucaacuccuccGacu...................	1	1	seq
....ugagauucaacuUcuccaacu...................	1	1	seq
....Ngagauucaacuccuccaacu...................	4	1	seq
....ugagauGcaacuccuccaacu...................	1	1	seq
....Ggagauucaacuccuccaacu...................	3	1	seq
....ugagauuUaacuccuccaacu...................	3	1	seq
....ugagauucaacuccucUaacu...................	9	1	seq
....ugagGuucaacuccuccaacu...................	2	1	seq
....ugagauucaacuccuUcaacu...................	1	1	seq
....uUagauucaacuccuccaacu...................	1	1	seq
....ugagauucaaAuccuccaacu...................	2	1	seq
....ugagCuucaacuccuccaacu...................	1	1	seq
....ugagauucUacuccuccaacu...................	1	1	seq
....ugagauucaacuccuccaGcug.................	28	1	seq
....ugagauucaacuccAccaacug.................	21	1	seq
....ugagauucaacGccuccaacug.................	1	1	seq
....ugagauCcaacuccuccaacug.................	5	1	seq
....ugagauucaacuAcuccaacug.................	146	1	seq
....ugagauucaacuccuccCacug.................	4	1	seq
....ugagauucaCcuccuccaacug.................	3	1	seq
....uCagauucaacuccuccaacug.................	10	1	seq
....uNagauucaacuccuccaacug.................	2	1	seq
....ugagauucGacuccuccaacug.................	32	1	seq
....Ngagauucaacuccuccaacug.................	28	1	seq

Star **Mature**

```
uuucguucuuuuccguuugcgguggcuugagauuaguggagagaguuuuaucucaucgagcuugauuugggugagauucaacuccuccaacugcucucacacugaauuugc
.......................................................................ugagauucaacuccuccaacAg..................  26      1  seq
.......................................................................ugagauucaacuccGccaacug..................  10      1  seq
.......................................................................Agagauucaacuccuccaacug..................  280     1  seq
.......................................................................ugaCauucaacuccuccaacug..................  6       1  seq
.......................................................................ugagauucaacuccuccaacug..................  109601  0  seq
.......................................................................ugagauucaacuccuGcaacug..................  5       1  seq
.......................................................................ugagauAcaacuccuccaacug..................  38      1  seq
.......................................................................ugagaGucaacuccuccaacug..................  6       1  seq
.......................................................................ugNgauucaacuccuccaacug..................  1       1  seq
.......................................................................ugCgauucaacuccuccaacug..................  5       1  seq
.......................................................................ugagauucaacCccuccaacug..................  5       1  seq
.......................................................................ugGgauucaacuccuccaacug..................  13      1  seq
.......................................................................ugagauucaacucAuccaacug..................  72      1  seq
.......................................................................ugUgauucaacuccuccaacug..................  5       1  seq
.......................................................................ugagauucaacuGcuccaacug..................  7       1  seq
.......................................................................ugagauucaaAuccuccaacug..................  7       1  seq
.......................................................................ugagaAucaacuccuccaacug..................  25      1  seq
.......................................................................Ggagauucaacuccuccaacug..................  26      1  seq
.......................................................................ugagauucaacuccuccaacuU..................  22      1  seq
.......................................................................ugagauucaacuccuccaacuA..................  13      1  seq
.......................................................................ugagaCucaacuccuccaacug..................  9       1  seq
.......................................................................ugagauuAaacuccuccaacug..................  73      1  seq
.......................................................................ugagauucaacuccucAaacug..................  35      1  seq
.......................................................................ugagauucaNcuccuccaacug..................  1       1  seq
.......................................................................ugagauucNacuccuccaacug..................  1       1  seq
.......................................................................uUagauucaacuccuccaacug..................  6       1  seq
.......................................................................ugagauucaacuccuAcaacug..................  18      1  seq
.......................................................................ugagauucaacuUcuccaacug..................  35      1  seq
.......................................................................ugagCuucaacuccuccaacug..................  4       1  seq
.......................................................................ugagauucCacuccuccaacug..................  5       1  seq
.......................................................................ugagGuucaacuccuccaacug..................  11      1  seq
.......................................................................ugagauucaacuccuUcaacug..................  23      1  seq
.......................................................................ugagauucaacucGuccaacug..................  1       1  seq
.......................................................................ugagauucaUcuccuccaacug..................  15      1  seq
.......................................................................ugaAauucaacuccuccaacug..................  10      1  seq
.......................................................................ugagUuucaacuccuccaacug..................  9       1  seq
.......................................................................ugagauucaacuccuccaacNg..................  1       1  seq
.......................................................................ugagauucaacuccuccaUcug..................  12      1  seq
.......................................................................ugagauuGaacuccuccaacug..................  5       1  seq
.......................................................................ugagauucaacuccucGaacug..................  7       1  seq
.......................................................................ugagaNucaacuccuccaacug..................  6       1  seq
.......................................................................ugagauucaacuccuccaCcug..................  2       1  seq
.......................................................................ugagauucaacuccuccaaUug..................  5       1  seq
.......................................................................ugagauucaaGuccuccaacug..................  2       1  seq
.......................................................................ugagauucaacuccuccaacCg..................  9       1  seq
.......................................................................ugagauucaacAccuccaacug..................  38      1  seq
.......................................................................ugagauucaaUuccuccaacug..................  11      1  seq
.......................................................................ugagauucaacuccucUaacug..................  163     1  seq
.......................................................................ugagauuUaacuccuccaacug..................  74      1  seq
.......................................................................ugagauucaacuccuccaacGg..................  400     1  seq
.......................................................................ugagauucaacuccCccaacug..................  44      1  seq
.......................................................................ugagauucaacuccuccGacug..................  21      1  seq
.......................................................................ugagauucaacuccuccaaAug..................  33      1  seq
.......................................................................ugagauucaGcuccuccaacug..................  21      1  seq
.......................................................................uAagauucaacuccuccaacug..................  99      1  seq
.......................................................................ugaUauucaacuccuccaacug..................  3       1  seq
.......................................................................ugagauucaacucUuccaacug..................  120     1  seq
.......................................................................ugagauucaacuccuccUacug..................  6       1  seq
.......................................................................ugagauGcaacuccuccaacug..................  3       1  seq
.......................................................................ugagauucaacuccuccaaGug..................  2       1  seq
.......................................................................Cgagauucaacuccuccaacug..................  19      1  seq
.......................................................................ugagauucUacuccuccaacug..................  11      1  seq
.......................................................................ugagauucaacuccuccaacuC..................  13      1  seq
.......................................................................ugagauucaaUuccuccaacugc.................  8       1  seq
.......................................................................ugagauucaacucUuccaacugc.................  89      1  seq
.......................................................................ugagauuUaacuccuccaacugc.................  143     1  seq
.......................................................................ugagauucaacuccucGaacugc.................  20      1  seq
.......................................................................ugagCuucaacuccuccaacugc.................  4       1  seq
.......................................................................ugagauucaacuccuccaacAgc.................  71      1  seq
.......................................................................Ngagauucaacuccuccaacugc.................  65      1  seq
```

Star Mature

uuucguucuuuuccguuugcggugcuugagauuaguggagagaguuuuaucucaucgagcuugauuugggugagauucaacuccuccaacugcucucacacugaauuugc

....ugagauucUacuccuccaacugc.................	14	1	seq
....Ggagauucaacuccuccaacugc.................	74	1	seq
....ugagGuucaacuccuccaacugc.................	20	1	seq
....ugagauucaacuccuccUacugc.................	12	1	seq
....uCagauucaacuccuccaacugc.................	18	1	seq
....Agagauucaacuccuccaacugc.................	482	1	seq
....ugagauucGacuccuccaacugc.................	64	1	seq
....ugagauAcaacuccuccaacugc.................	65	1	seq
....ugagauucaacuccuccaacuAc.................	23	1	seq
....ugagauucCacuccuccaacugc.................	4	1	seq
....ugagauucaGcuccuccaacugc.................	34	1	seq
....ugagauucaacuccuccGacugc.................	39	1	seq
....ugagauucaacCccuccaacugc.................	24	1	seq
....ugagauucaacuccuccaCcugc.................	2	1	seq
....ugagauCcaacuccuccaacugc.................	15	1	seq
....ugagauucaacuUcuccaacugc.................	17	1	seq
....ugagauucaacuccuccaacugc.................	180958	0	seq
....ugagauucaacuccuccaacugN.................	2	1	seq
....ugagauucaacuccuccaUcugc.................	11	1	seq
....uNagauucaacuccuccaacugc.................	4	1	seq
....ugagauucaacuccCccaacugc.................	44	1	seq
....ugagauucaacuccuccaaAugc.................	28	1	seq
....Cgagauucaacuccuccaacugc.................	38	1	seq
....ugaUauucaacuccuccaacugc.................	10	1	seq
....ugagauucaacuccuUcaacugc.................	44	1	seq
....ugagauucaacuccuccaacugU.................	56807	1	seq
....ugagauucaCcuccuccaacugc.................	5	1	seq
....ugagauucaacucGuccaacugc.................	3	1	seq
....ugagauucaacuGcuccaacugc.................	15	1	seq
....ugagauucaacuccuccaacugG.................	178	1	seq
....ugagauucaacuccuAcaacugc.................	35	1	seq
....ugagaNucaacuccuccaacugc.................	4	1	seq
....uAagauucaacuccuccaacugc.................	213	1	seq
....ugUgauucaacuccuccaacugc.................	23	1	seq
....uUagauucaacuccuccaacugc.................	13	1	seq
....ugagauucaacuccuccaacNgc.................	1	1	seq
....ugagauucaacuccuccaacGgc.................	6	1	seq
....ugagauucaacGccuccaacugc.................	4	1	seq
....ugagauucaacuccuGcaacugc.................	8	1	seq
....ugaAauucaacuccuccaacugc.................	20	1	seq
....ugagauucaacuccuccaacuCc.................	12	1	seq
....ugGgauucaacuccuccaacugc.................	34	1	seq
....ugagauucaacAccuccaacugc.................	67	1	seq
....ugagauucaacuccuccaaUugc.................	7	1	seq
....ugagauucaacuccuccaacCgc.................	25	1	seq
....ugagauucaNcuccuccaacugc.................	4	1	seq
....ugCgauucaacuccuccaacugc.................	2	1	seq
....ugagauucaaAuccuccaacugc.................	19	1	seq
....ugagauucaacuccuccCacugc.................	2	1	seq
....ugagaAucaacuccuccaacugc.................	52	1	seq
....ugagauucaaNuccuccaacugc.................	1	1	seq
....ugagauucaaGuccuccaacugc.................	4	1	seq
....ugagauucaUcuccuccaacugc.................	23	1	seq
....ugagauucaacuccuccaacuUc.................	13	1	seq
....ugagUuucaacuccuccaacugc.................	13	1	seq
....ugagauucaacuccucUaacugc.................	294	1	seq
....ugaCauucaacuccuccaacugc.................	9	1	seq
....ugagauucNacuccuccaacugc.................	1	1	seq
....ugagaCucaacuccuccaacugc.................	16	1	seq
....ugagauucaacucAuccaacugc.................	109	1	seq
....ugNgauucaacuccuccaacugc.................	2	1	seq
....ugagauGcaacuccuccaacugc.................	13	1	seq
....ugagauuAaacuccuccaacugc.................	92	1	seq
....ugagauucaacuccuccaaGugc.................	1	1	seq
....ugagauucaacuccAccaacugc.................	48	1	seq
....ugagauucaacuAcuccaacugc.................	88	1	seq
....ugagauuGaacuccuccaacugc.................	10	1	seq
....ugagauucaacuccuccaacugA.................	421	1	seq
....ugagaGucaacuccuccaacugc.................	8	1	seq
....ugagauucaacuccucAaacugc.................	50	1	seq

Star Mature

uuucguucuuuuccguuugcggugcuugagauuaguggagagaguuuuaucucaucgagcuugauuugggugagauucaacuccuccaacugcucucacacugaauuugc			
....ugagauucaacuccGccaacugc.................	4	1	seq
....ugagauucaacuccuccaGcugc.................	23	1	seq
....Agagauucaacuccuccaacugcu................	2	1	seq
....ugagauuGaacuccuccaacugcu................	1	1	seq
....ugagauucaacuccuccaacugAu................	5	1	seq
....ugagauucaacuccucAaacugcu................	4	1	seq
....ugagauucaacuccuccaacugcC................	5	1	seq
....ugagauuAaacuccuccaacugcu................	2	1	seq
....ugagauucaacuccuccaacugcA................	222	1	seq
....ugagauucaacucAuccaacugcu................	2	1	seq
....ugagauucaacuccuccaGcugcu................	2	1	seq
....ugagauucaacuccuccaacuAcu................	3	1	seq
....ugaAauucaacuccuccaacugcu................	2	1	seq
....uAagauucaacuccuccaacugcu................	4	1	seq
....ugagauAcaacuccuccaacugcu................	2	1	seq
....uCagauucaacuccuccaacugcu................	1	1	seq
....ugagauucGacuccuccaacugcu................	3	1	seq
....ugagauucaacuccuccaacugcu................	2951	0	seq
....ugagauucaacuccuccaacuUcu................	1	1	seq
....ugagauucaacuccucUaacugcu................	5	1	seq
....Ngagauucaacuccuccaacugcu................	1	1	seq
....ugagauucaacuccuccaacAgcu................	5	1	seq
....ugagauucaacuccAccaacugcu................	1	1	seq
....ugagauucaacuccuccaacugcG................	10	1	seq
....uUagauucaacuccuccaacugcu................	1	1	seq
....ugagauucaacuccuccaacugUu................	751	1	seq
....ugagauucaacuccuccaacugcuG...............	11	1	seq
....ugagauucaacuccuccaacugcuU...............	133	1	seq
....ugagauucaacuccuccaacugcuUu..............	9	1	seq
....gagauucaacuccuccaac....................	1	0	seq
....gagauucaacuccuccaacu...................	2	0	seq
....gagauucaaAuccuccaacug..................	1	1	seq
....gagauucaacuccGccaacug..................	1	1	seq
....gagauucaacuccuccaacAg..................	2	1	seq
....gagauucaCcuccuccaacug..................	1	1	seq
....gagauucCacuccuccaacug..................	1	1	seq
....gagauucaacuAcuccaacug..................	1	1	seq
....gagauucaacuccAccaacug..................	1	1	seq
....gagauucaacuccuccaacug..................	138	0	seq
....Uagauucaacuccuccaacugc.................	1	1	seq
....gagauucaacuccuAcaacugc.................	1	1	seq
....gagauuGaacuccuccaacugc.................	1	1	seq
....gagauucaCcuccuccaacugc.................	1	1	seq
....gagauucaGcuccuccaacugc.................	1	1	seq
....gagauucaacuccuccaacugc.................	204	0	seq
....gagauucaacuAcuccaacugc.................	3	1	seq
....gagauucaacAccuccaacugc.................	5	1	seq
....gagauucaacuccuccaacugU.................	40	1	seq
....gagauucaacuccuccaacAgc.................	5	1	seq
....Ggauucaacuccuccaacug..................	1	1	seq
....Ugauucaacuccuccaacug..................	1	1	seq
....agauucaacuccuccaacug..................	72	0	seq
....agauucaacuccAccaacugc.................	1	1	seq
....agauucaacuccuccaacugc.................	112	0	seq
....Ugauucaacuccuccaacugc.................	7	1	seq
....agauucaacuccuccaacugU.................	1	1	seq
....agauuAaacuccuccaacugc.................	1	1	seq
....agaAucaacuccuccaacugc.................	1	1	seq
....agauucaacuccuccaacugG.................	1	1	seq
....gauucaacuccuccaacug..................	25	0	seq
....gauucaacuccuccaacGg..................	1	1	seq
....gauucaacuccuccaacugc.................	38	0	seq
....gauucaacuccuccaacugU.................	7	1	seq
....gaCucaacuccuccaacugc.................	1	1	seq
....auucaacuccuccaacug..................	27	0	seq
....auucaacuccuccaacugc.................	167	0	seq
....auucaacuccuccaacugU.................	26	1	seq
....Guucaacuccuccaacugc.................	1	1	seq
....auCcaacuccuccaacugc.................	1	1	seq
....auucaacuAcuccaacugc.................	1	1	seq

```
                                Star                                 Mature
uuucguucuuuuccguuugcggugcuugagauuaguggagagaguuuuaucucaucgagcuugauuuggguaugagauucaacuccuccaacugcucucacacugaauuugc
...........................................................................uucaacuccuccaacugc.................    53    0    seq
...........................................................................uucaacuccuccaacugcu................    1     0    seq
............................................................................ucaacuccuccaacugcucucacacugaauu...    1     0    seq
```
